# A novel investigation of the effect of iterations in sliding semi-landmarks for 3D human facial images

**DOI:** 10.1186/s12859-020-3497-7

**Published:** 2020-05-24

**Authors:** Azree Nazri, Olalekan Agbolade, Razali Yaakob, Abdul Azim Ghani, Yoke Kqueen Cheah

**Affiliations:** 1 Department of Computer Science, Faculty of Computer Science & IT, Universiti Putra Malaysia, Selangor, Malaysia; 2 Institute of Bioscience, Universiti Putra Malaysia, Selangor, Malaysia; 3 Department of Software Engineering, Faculty of Computer Science & IT, Universiti Putra Malaysia, Selangor, Malaysia; 4 Department of Biomedical Science, Faculty of Medicine and Health Sciences, Universiti Putra Malaysia, Selangor, Malaysia

**Keywords:** Facial landmarks, Sliding semi-landmarks, 3D faces, Multi-point warping, PCA, LDA

## Abstract

**Background:**

Landmark-based approaches of two- or three-dimensional coordinates are the most widely used in geometric morphometrics (GM). As human face hosts the organs that act as the central interface for identification, more landmarks are needed to characterize biological shape variation. Because the use of few anatomical landmarks may not be sufficient for variability of some biological patterns and form, sliding semi-landmarks are required to quantify complex shape.

**Results:**

This study investigates the effect of iterations in sliding semi-landmarks and their results on the predictive ability in GM analyses of soft-tissue in 3D human face. Principal Component Analysis (PCA) is used for feature selection and the gender are predicted using Linear Discriminant Analysis (LDA) to test the effect of each relaxation state. The results show that the classification accuracy is affected by the number of iterations but not in progressive pattern. Also, there is stability at 12 relaxation state with highest accuracy of 96.43% and an unchanging decline after the 12 relaxation state.

**Conclusions:**

The results indicate that there is a particular number of iteration or cycle where the sliding becomes optimally relaxed. This means the higher the number of iterations is not necessarily the higher the accuracy.

## Background

Geometric Morphometrics approach differs from traditional morphometrics because it was predicated on the statistical theory of shape and utilizes geometric information collected through the landmark coordinates. This produces various powerful and flexible statistical procedures for shape investigation [1], which are directly interpreted using graphic visualizations [2]. The data obtained during landmarks acquisition follow homology rules, such that, all landmarks must be the same number and be positioned in the same order throughout the specimen. These landmarks can be replicated from subject to subject based on common geometry, common function, and common morphology [3]. With the introduction of Thin Plate-Spline (TPS) and Iterative Closest Point (ICP), the corresponding landmarks of the beginning and target form appear precisely in the corresponding positions and landmark correspondence can now be repeatedly registered in the neighborhood of a landmark [4, 5].

GM of landmarks of 2D or 3D coordinates are the most commonly applied in morphometrics [5, 6]. Through the projection from a reference form, semi-landmarks could be placed semi-automatically by estimating the positions on the target surface [7]. This makes the application of semi-landmarks in geometric analysis of curves and surfaces easy, providing more precise quantification structure which are not available for classical landmark-based [8]. In landmark-based geometric analysis, after the differences due to location, scale and orientation are removed, with the help of Generalized Procrustes Analysis (GPA) [9, 10], shape can be defined as the information remaining in a configuration of landmark points [11, 12]. This produces a set of subjects in partial Procrustes superimposition with respect to a common reference form [13, 14].

Facial landmarking is a crucial step in the facial analysis for biometrics and numerous other applications. Because 3D data contain more information and are less sensitive to illumination and occlusion than that of 2D, the use of 3D data to improve facial analysis has become a trend in computer vision [15]. Consequently, in the course of extracting facial features for facial analysis, the problem of landmarks has been extensively studied in faces. In [16], a literature of some algorithms were presented based on occlusion detection which may be provoked by hair or external objects; such as hats, glasses, scarves, or by the subject’s hands. Hence, due to the decrease in performance when facial area is partially occluded, facial changes are important factors to be considered by face recognition systems [16]. A novel automatic method for facial landmark localization was proposed in [17] to improve performance recognition in human face. The method relies on geometrical properties of 3D facial surface that work on complete face by displaying different emotions in the presence of occlusions. A total of 8 anatomical landmarks (subnasale, pronasale, alare, nasion, endocanthion, exocanthion, inner and outer eyebrows) were selected one-by-one for the study. Under constant geometrical condition, the method double-checks to ensure alare, nasion, and pronasale are correctly localized, else the process starts afresh. A novel fusion pipeline was presented in [18] to address the problem of extreme head pose estimation from intensity images in a monocular setup. The method integrates and updates the covariance of Kalman Filter in every frame. A set of key-points is used to carry out tracking technique and extracts points in the head region. The method proved suitability cases with occlusions and extreme head rotations by relying on the alignment of facial landmarks in each frame.

Geometric analysis of curves and surfaces were made possible through the application of semi-landmarks. Where many structures cannot be quantified and larger areas of many biological objects cannot be captured using classical landmarks, semi-landmarks now provide a more precise quantification [19]. More so, it has been proven in [20] that only anatomical landmarks are insufficient to investigate shape variation of some biological patterns, thus, the method of sliding semi-landmarks was introduced. The sliding semi-landmarks were developed to be placed on surfaces or curves [8, 11] by minimizing bending energy [21, 22] or Procrustes distance [6, 23] which should be homologous throughout the subjects [24].

To perform the sliding of landmarks in 3D, several software packages are currently available. To name but a few, Edgewarp [25], EVAN toolbox (http://evan-society.org), Viewbox [26], Mathematica [23], and R packages: geomorph [27] and Morpho [28], etc.

Sliding semi-landmarks have been used in the study of bones surface such as articular and the diaphysis [22] and curves, providing descriptors of outlines and crests [29, 30]. In [6], sliding semi-landmarks were used to investigate craniofacial and dental variation in human by minimizing bending energy and Procrustes distance. Sliding semi-landmarks that are relaxed against a symmetrized mean were used in [31] to circumvent the problem of asymmetry caused by manual semi-landmarks, using bending energy to investigate nasal soft tissue reconstruction. To evaluate difference software packages for semi-landmark, sliding semi-landmarks were used in [20] to analyze the workflow complexity and time consumption to complete the sliding task and accounted for the duration to perform each task. However, asides this work, to the best of our knowledge, no study has presented any research in accounting for the time (duration) to perform the sliding of semi-landmark task for soft-tissue facial analysis. Furthermore, no study has investigated the effect of iteration in sliding semi-landmark for accuracy prediction.

**Fig. 1:**
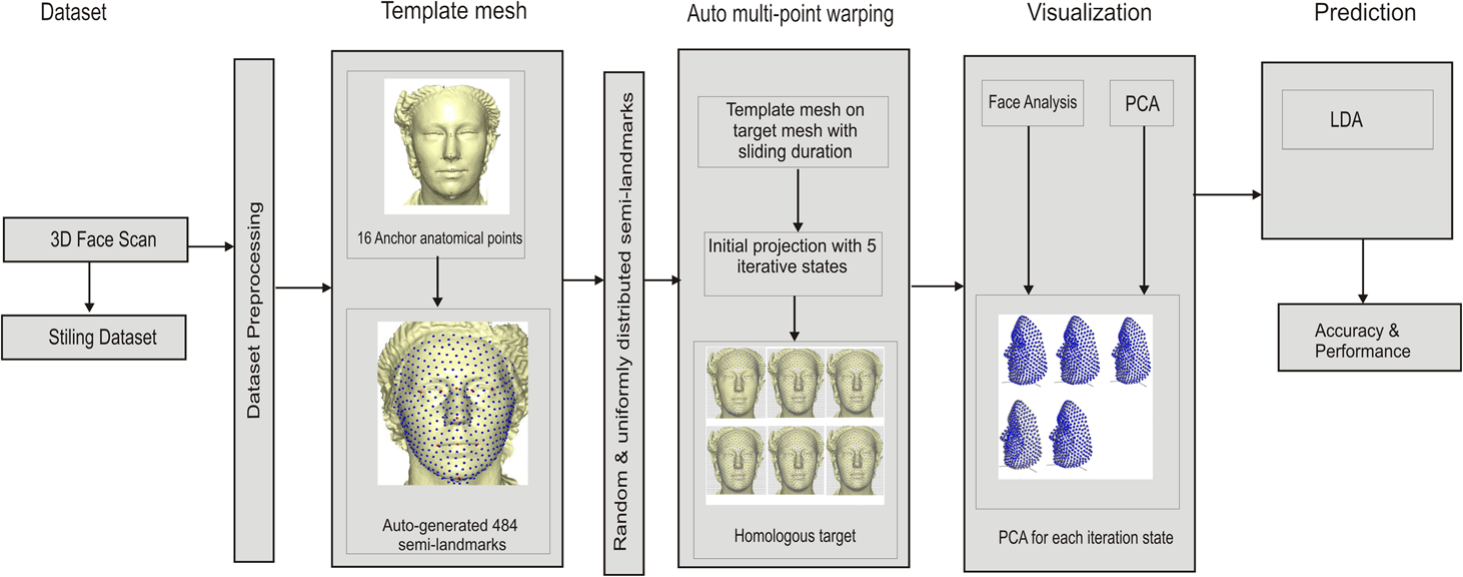
Architectural diagram of the application of multi-point warping for sliding iterations in 3D

This work aims to investigate whether the number of iteration in sliding semi-landmarks has effect on the predictive result or classification accuracy in geometric morphometric analyses of soft-tissue landmark-based in 3D human face. This is done by projecting the surface semi-landmarks from the template object to the target objects and iteratively sliding the semi-landmarks to a point relaxed. Here we used five relaxation states (one, six, twelve, twenty-four, and thirty) to ensure convergence and optimum smoothness. PCA was used as dimensionality reduction and feature selection due to the many number of facial points. The results for each relaxation state are further analyzed to predict the classification accuracy using LDA; and the visualization was performed using relative warp of the principal components. Figure 1 shows the architectural diagram of the application of multi-point warping for sliding iterations in 3D.

## Methods

### Dataset description

The dataset consists of 80 (40 males and 40 females) randomly selected sujects in wavefront obj format from Stirling/ESRC 3D Face Database [32]. The 3D facial scans which are intended to facilitate research in face recognition, expression recognition, sexual dimorphism, and perception were acquired in neutral position.

### Template mesh and target warping

**Fig. 2:**
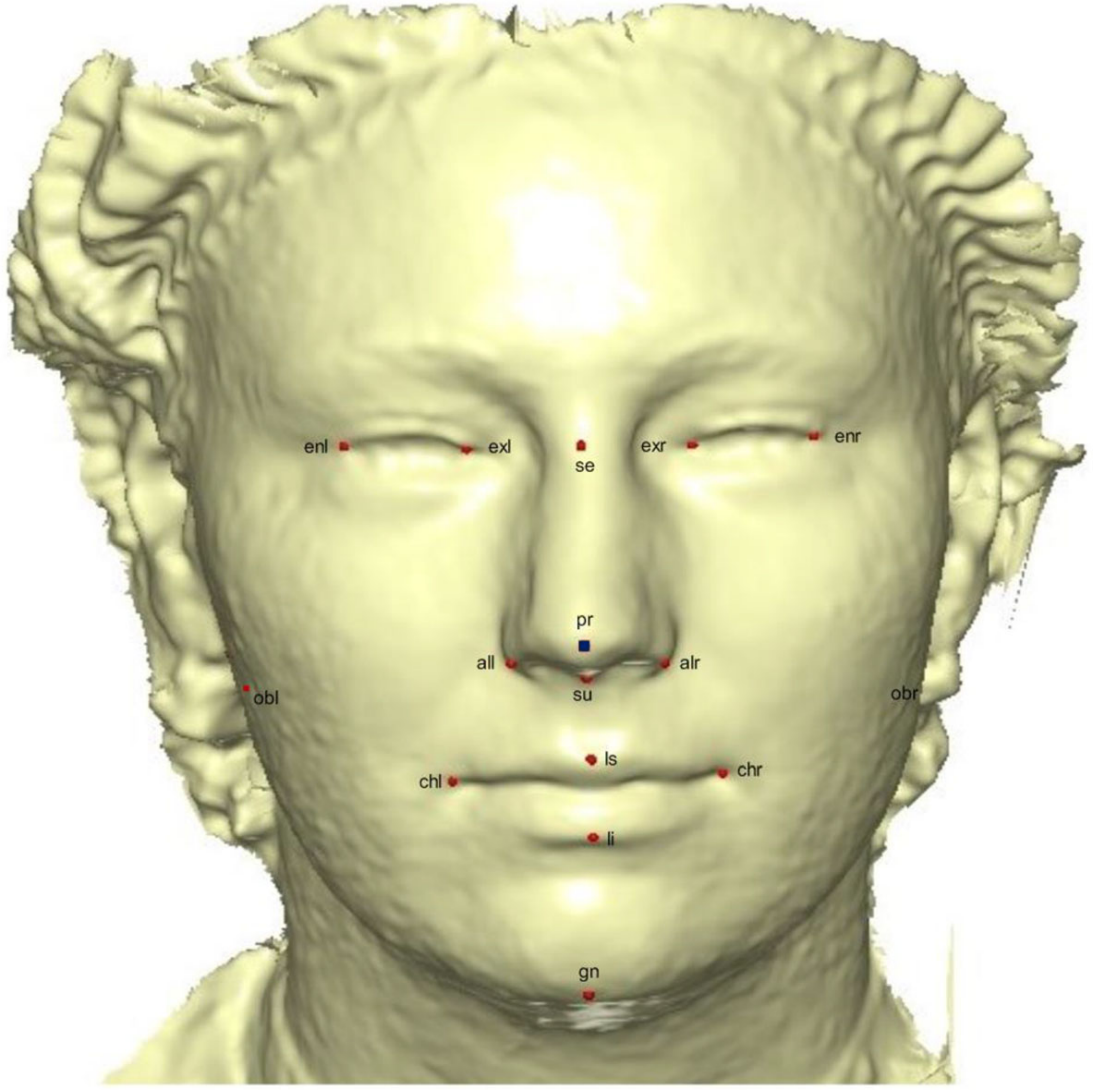
A three-dimensional mesh template with the location of the prominent point at the center of the face for pose-invariant correction. The 16 fixed anatomical landmarks are shown in red color. The blue color on the Pronasale indicates the point at which the semi-landmarks begin the sliding process

To build the template, we manually located 16 anatomical points on a 3D mesh (Fig. 2), following the landmark standard in [33, 34]. The landmarks description can be found in [35]. The modeling of the template and sliding of semi-landmarks was performed in Viewbox 4.0 [26] using geometric morphometric tools based on the methodology in [35–38]. The anchor anatomical points were not subjected to sliding but were used to build the warping fields for the minimization of bending energy. We chose the pronasale to begin the sliding process because of its invariance to facial expression, pose correction and easy detection [39, 40]. Using this point, 484 semi-landmark were automatically generated which overlapped on the pronasale point and later uniformly spread on the facial surface with 1.5 mm radius. This was chosen to accomodate all the 500 points.

**Fig. 3:**
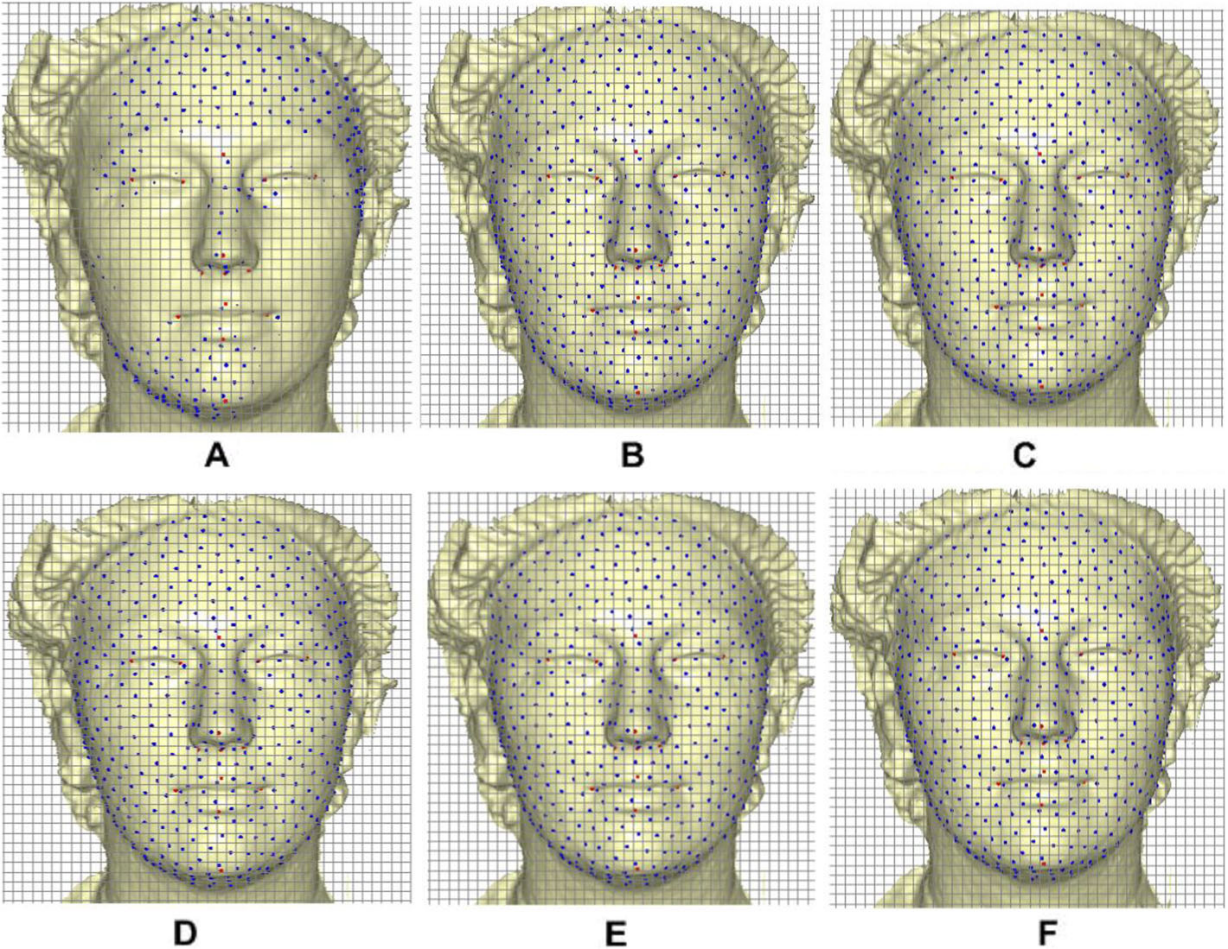
Sliding point warped on target facial surface. **a** Initia projection. **b** One iteration. **c** Six iterations. **d** Twelve iterations. **e** Twenty-four iterations. **f** Thirty iterations

By applying the TPS warping, the semi-landmarks slid along the curves and surfaces of the mesh on each target by minimizing the bending energy. The process went through different iterative steps based on the five relaxation states (Fig. 3) to optimally and homologously relax the sliding points. Minimization of the bending energy makes the sliding points homologous to the template configuration. See the studies in [35, 36] for detailed implementation of the sliding and warping tasks.

### Time estimation

The time for initial surface projection, sliding of each set of iterations against Procrustes consensus was measured using a timer. All analyses were run on a desktop computer (Dell Optiplex 7010) with a Intel® Core i5–3470 CPU, 8Gb of memory, running on Windows 7 Professional (64 bit).

### Feature selection, visualization and Clasification

The data analysis, visualization and classification were performed using PAST 3.0 [41]. The features are selected by dimensionality reduction using Principal Components Analysis (PCA). The PCA yielded 97PCs in total and we chose the first 30PCs which have the highest ranking eigenvectors for each set which accounted for more than 95% variance. But for the visualization of the change in variation, the first PC of the relative warp which accounted for the highest variation was plotted. Linear Discriminant Analysis (LDA) was used to classify the selected PCs since it is easy to implement and no parameter tuning or adjustment required. It has been successfully used in the previous studies to classify gender in morphometrics [42–44].

## Results

### Time estimation

The times measured for surface projection and iterative sliding for relaxation for each set against Procrustes mean shape are presented in Table 1. The time patterns indicate that the higher the number of iterations the longer the processing time.

**Table 1:** Duration of each tasks of the semi-landmark sliding procedure on the whole dataset

Sliding	Time
Initial Projection (s)	480
1 Iteration (s)	144
6 Iterations (s)	3040
12 Iterations (min)	96
24 Iterations (min)	160
30 Iterations (min)	320

### PCA visualization and classification

**Fig. 4:**
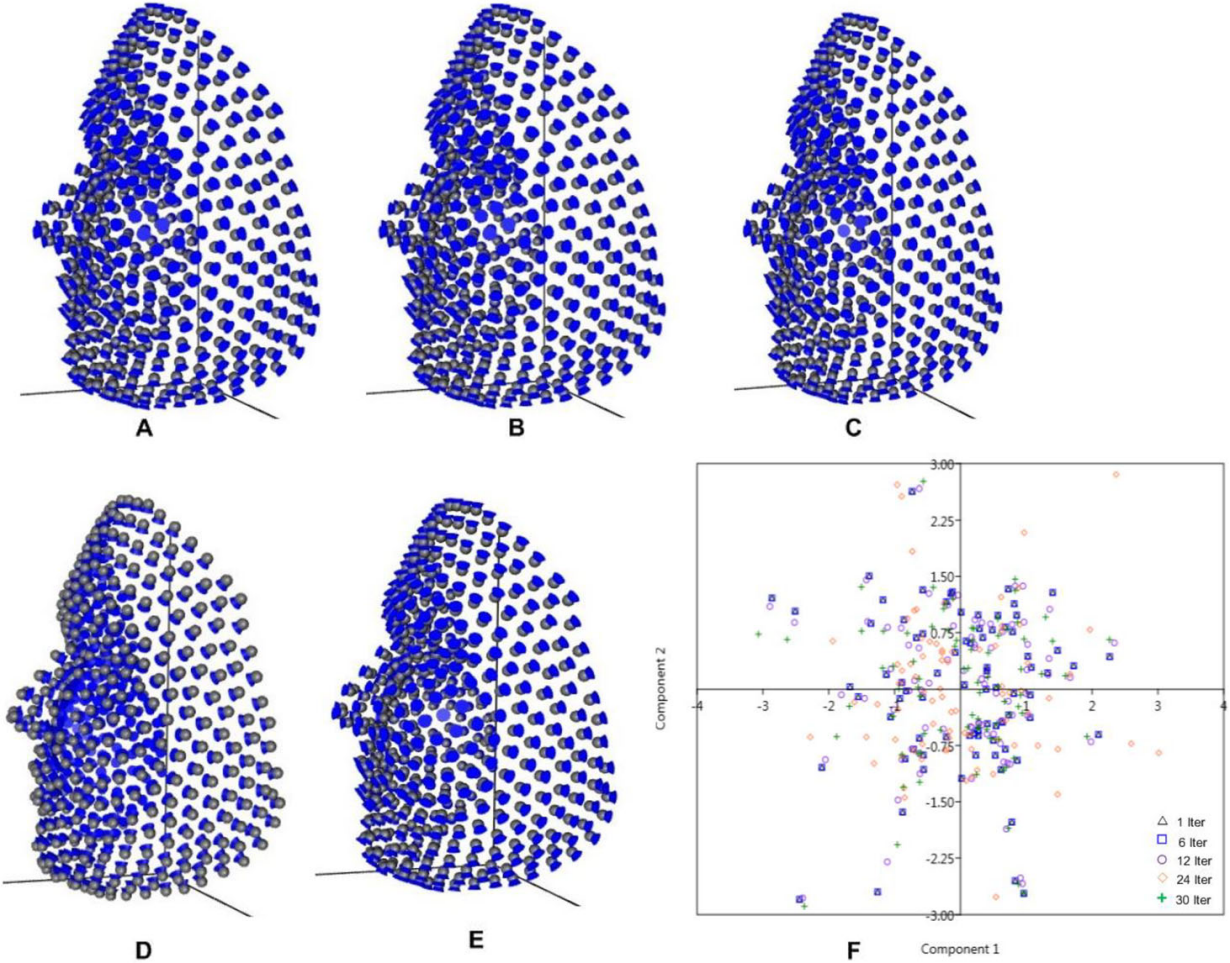
Relative warp of the first principal component of each sliding set and Scatterplot. **a** One iteration. **b** Six iterations. **c** Twelve iterations. **d** Twenty-four iterations. **e** Thirty iterations. **f** Scatterplot of the first two principal components

The first 2PCs of the PCA explain more than 50% of the variance for each sliding set: one iteration (PC1 = 34.15%, PC2 = 19.79%); six iterations (PC1 = 34.15%, PC2 = 19.79%); twelve iterations (PC1 = 34.41%, PC2 = 18.42%); twenty-four iterations (PC1 = 33.99%, PC2 = 18.30%); and thirty iterations (PC1 = 33.58%, PC2 = 18.46%). To visualise the pattern of variation in each relaxation state of the iterative sliding, the relative warp of the first principal component of each sliding set is presented (Fig. 4a-3e); alongside with the scatterplot of the PC1 vs PC2 (Fig. 4f), showing the distribution of specimens in morphological space. The selected PC scores are subjected to LDA to predict the gender in PAST software for each sliding set as shown in Table 2 and the gender were maximally discriminated.

**Table 2:** Accuracy of each relaxation state using LDA

Iteration cycle	Accuracy (%)
1 Iteration	94.64
6 Iterations	94.64
12 Iterations	96.43
24 Iterations	92.86
30 Iterations	92.86

## Discussion

In this study, sliding semi-landmarks iteration was investigated. The approach minimized bending energy on a set of five iterative states (one, six, twelve, twenty-four, and thirty). The sliding task was necessary for the comparison of shapes and forms. Because the use of manual semi-landmarks may not be appropriate to measure shape variation when surfaces and curves on the target are not homologous. Each iterative state task was performed separately. The duration for subsequent iterative relaxation operations for each iterative state was computed and presented. The timing follows a consecutively progressive patterns such that the initial surface projection takes 480 s, one iteration takes 144 s, six iterations takes 3040, twelve iterations takes 96 min, twenty-four iterations takes 160 min, and thirty iterations takes 320 min. To the best of our knowledge, duration in sliding semi-landmark for soft-tissue 3D in human face has not been proposed in any literature asides the work presented in [20], where time difference was computed and analysed between two software packages (Morpho and Edgewarp) for the same sliding tasks.

The principal components between relaxation state one and relaxation state six show no observable difference in variation (PC1 = 34.15%, PC2 = 19.79%), both contains exactly the same percentage variances throughout the PC variables. Meanwhile, there is a slight difference in percentage variance in other relaxation states; though a noticeable decline occurs in PC1 from twelve relaxation state to thirty relaxation state. The relative warp visualisations show no observable difference between one and six relaxation states. But there is observable difference among twelve, twenty-four and thirty relaxation states. The distribution of specimens in morphological space shows a strong overlapping for one and six relaxation states. Both the black triangles and the blue square boxes sit on each other in the morphospace. But there is observable spread among the rest relaxation states.

Using LDA, the gender was maximally discriminated [45]. This was applied to test the accuracy of each relaxation state and possibly answer the hypothesis question, “does the number of iteration in sliding semi-landmarks have effect on the predictive result or classification accuracy?” The first two states (one and six) have exactly equal accuracy (94.64%); meaning that, the number of cycles has no effect on both states. Same is observed for twenty-four and thirty states, having exactly the same accuracy (92.86%). We noticed stability at twelve relaxation state with highest accuracy of 96.43% and a constant decline after the twelve relaxation state. This is an indication that the higher the number of cycles does not in any way indicate the higher the classification accuracy. It also means that there is a particular number of iteration or cycle where the sliding becomes optimally relaxed.

The model performance was measured using precision, sensitivity and specificity while the dataset was divided into 70% training and 30% testing, as no parameter turning is required in LDA. Table 3 presented the performance metric reports with 12 iteration state having the highest sensitivity (0.961) and specificity (0.966). This is an indication that the sliding process is best relaxed at twelve iterations when the bending energy was minimized.

**Table 3:** Performance metrics reports for the five sliding states using LDA

Iteration	Precision	Sensitivity	Specificity
1 Iteration	0.961	0.925	0.965
6 Iterations	0.961	0.925	0.965
12 Iterations	0.961	0.961	0.966
24 Iterations	0.923	0.923	0.933
30 Iterations	0.923	0.923	0.933

## Conclusions

In conclusion, this study investigates the predictive ability of sliding semi-landmarks using various iterative states and duration of time requires in performing the sliding tasks for each state. However, it is observed that the classification accuracy was affected by the number of iterations but not in progressive pattern (i.e. the higher the number of iterations is not necessarily the higher the accuracy). This study is based on Stirling/ERSC dataset which is European population, therefore the methods and results proposed may be tested in other populations. Furthermore, there is a noticeable challenge in the annotation of the eyeball in Viewbox 4.0 when the eyes are opened. Though, it does not affect the annotation of endocanthion and exocanthion. This will be looked into in our future studies.

## Supplementary information

**Additional file 1.**Three-dimensional raw data for each iteration state.

**Additional file 2.** PCs scores for each iteration state.

## Data Availability

Raw three-dimensional digitized data and principal component analysis scores for each iteration state. Table S1: Three-dimensional raw data for each iteration state, Table S2: PCs scores for each iteration state. Note that the 3D face dataset is not permitted to be shared by third party according to the license agreement.

## Abbreviations

GPA: General Procrustes Analysis
GM: Geometric Morphometric
PCA: Principal Component Analysis
LDA: Linear Discriminant Analysis
TPS: Thin Plate-Spline
PAST: Paleontological Statistics Software
